# The Cholesteryl Ester Transfer Protein Inhibitor, des-Fluoro-Anacetrapib, Prevents Vein Bypass-induced Neointimal Hyperplasia in New Zealand White Rabbits

**DOI:** 10.1038/s41598-019-52510-0

**Published:** 2019-11-07

**Authors:** Ben J. Wu, Yue. Li, Kwok-Leung Ong, Yidan Sun, Douglas Johns, Philip J. Barter, Kerry-Anne Rye

**Affiliations:** 1 Lipid Research Group, School of Medical Sciences, The University of New South Wales Sydney, New South Wales, Australia; 2 Otto Loewi Research Center for Vascular Biology, Immunology and Inflammation, Immunology and Pathophysiology, Medical University of Graz, Graz, Austria; 3 Merck & Co., Inc, Kenilworth, NJ USA

**Keywords:** Lipoproteins, Carotid artery disease

## Abstract

Coronary artery bypass grafting is among the most commonly performed of all cardiovascular surgical procedures. However, graft failure due to stenosis reduces the long-term benefit of the intervention. This study asks if elevating plasma high density lipoprotein cholesterol (HDL-C) levels by inhibition of cholesteryl ester transfer protein (CETP) activity with des-fluoro-anacetrapib, an analog of the CETP inhibitor anacetrapib, prevents vein bypass-induced neointimal hyperplasia. NZW rabbits were placed on a normal chow diet or chow containing 0.14% (wt/wt) des-fluoro-anacetrapib for 6 weeks. Bypass grafting of the jugular vein to the common carotid artery was performed 2 weeks after starting dietary des-fluoro-anacetrapib supplementation. The animals were euthanised 4 weeks post-bypass grafting. Relative to control, dietary supplementation with des-fluoro-anacetrapib reduced plasma CETP activity by 89 ± 6.9%, increased plasma apolipoprotein A-I levels by 24 ± 5.5%, increased plasma HDL-C levels by 93 ± 26% and reduced intimal hyperplasia in the grafted vein by 38 ± 6.2%. Des-fluoro-anacetrapib treatment was also associated with decreased bypass grafting-induced endothelial expression of vascular cell adhesion molecule-1 (VCAM-1) and intercellular adhesion molecule-1 (ICAM-1), endothelial dysfunction, and smooth muscle cell (SMC) proliferation in the grafted vein. In conclusion, increasing HDL-C levels by inhibiting CETP activity is associated with inhibition of intimal hyperplasia in grafted veins, reduced inflammatory responses, improved endothelial function, and decreased SMC proliferation.

## Introduction

Coronary artery bypass grafting is among the most commonly performed of all cardiovascular surgical procedures. However, graft failure due to stenosis, which impairs blood flow, or can lead to total vessel occlusion, reduces the long-term benefit of this procedure. This is especially true for saphenous vein grafts.

Key factors in the pathogenesis of post-surgical vein graft occlusion are inflammatory responses that result in migration of inflammatory cells into the subendothelium of the vein grafts^1^, endothelial dysfunction due to decreased bioavailability of nitric oxide, increased endothelin-1 levels and enhanced superoxide production^2,3^. Smooth muscle cell proliferation and the transformation of smooth muscle cells in the grafted vessel wall from a quiescent, contractile phenotype to a proliferative, synthetic phenotype is also a common occurrence^4^.

Plasma high density lipoprotein cholesterol (HDL-C) levels are inversely correlated with the risk of having a cardiovascular event^5^. HDLs have multiple potentially cardioprotective properties. These include an ability to remove cholesterol from macrophages, which initiates the first step of the reverse cholesterol transport pathway^6,7^. HDLs also attenuate vascular inflammation^8^, suppress vascular smooth muscle cell (VSMC) proliferation^9^, promote endothelial repair^10^, and enhance endothelial function^11^. These cardioprotective functions of HDLs suggest that increasing endogenous HDL-C levels may reduce vein graft occlusion.

Cholesteryl ester transfer protein (CETP) transfers cholesteryl esters from HDLs to low density lipoproteins (LDLs) and triglyceride-rich lipoproteins in exchange for triglycerides^12^. Agents that inhibit CETP activity increase the level of HDL-C and apolipoprotein (apo) A-I, the major HDL apolipoprotein, and decrease non-HDL cholesterol levels. We have reported that the CETP inhibitor des-fluoro-anacetrapib, an analog of the CETP inhibitor anacetrapib, increases HDL-C levels^13^, enhances endothelial repair, improves endothelial function, inhibits vascular smooth muscle cell proliferation and reduces intimal hyperplasia in New Zealand White (NZW) rabbits following endothelial denudation of the abdominal aorta^13^ and in NZW rabbits with balloon injury and stent deployment in the iliac artery^14^.

The present study asks whether des-fluoro-anacetrapib treatment is associated with inhibition of neointimal hyperplasia in NZW rabbits with right external jugular vein autologous end-to-side transplantation bypass grafting in the right common carotid artery.

## Materials and Methods

### Animal studies

Two groups of male NZW rabbits (n = 8–9/group) weighing 2.5–3.0 kg (Nanowie Small Animal Production Unit, Bellbrae, Victoria, Australia) were maintained on a regular chow diet, or chow supplemented with 0.14% (wt/wt) des-fluoro-anacetrapib (Merck & Co., Inc. Kenilworth, NJ, USA) for 6 weeks.

After 2 weeks of des-fluoro-anacetrapib treatment, vein bypass grafting was performed under general anaesthesia (inhaled isofluorane (4–5% for induction and 1.5–2% for maintenance). A front midline neck incision was made to expose the right external jugular vein and the right common carotid artery in the anaesthetised animals. The branches of the jugular vein were carefully ligated with 8–0 polypropylene sutures. An approximately 2 cm segment of the jugular vein was removed, flushed and kept moist in heparinized saline (100U heparin/mL) for the autologous, reversed vein graft. The animals were systemically heparinized (200 IU/kg), and the right common carotid artery was clamped at the proximal and distal ends. The side of the artery was flushed with heparinized saline (1U heparin/mL) containing 1% (w/v) lignocaine. An 8/0 prolene uninterrupted suture was used to make a reversed vein attachment end-to-side to the artery (proximal end of the vein to the distal part of the artery and vice versa) under 3.5X magnification (Zeiss, Germany). The artery clamps were removed after graft anastomosis, and blood flow was restored into the grafted vein. The incision was closed with 4/0 silk suture, and the sutured skin was covered with iodine spray and wound gel (antiseptic). Pulsatile flow in the grafted vessel was confirmed by palpation. The animals were euthanised 4 weeks after bypass grafting, and the grafted vein and blood were collected. All the procedures were approved by the University of New South Wales Sydney Animal Care and Ethics Committee (Protocol number 15/144A) and performed in accordance with the relevant guidelines and regulations.

### Biochemical analyses

Blood was collected into EDTA tubes (BD Biosciences, Franklin Lakes, NJ) following euthanasia and plasma was isolated (1,000 × *g*, 4 °C, 10 min). CETP activity was assessed as the transfer of [^3^H]-labelled cholesteryl esters from ultracentrifugally isolated human HDL_3_ to isolated human LDLs^15^. This was achieved by incubation of plasma with [^3^H]cholesteryl ester-labelled HDL_3_ and unlabelled LDLs at 37 °C for 3 h. The LDLs were precipitated with heparin (5,000 IU/mL):MnCl_2_ (2 mol/L) (1:1, v/v) and the radioactivity in the supernatant was determined by liquid scintillation counting. The activity of CETP was determined as the % total radiolabelled cholesteryl esters transferred from HDL_3_ to LDLs^15^. ApoA-I concentrations were determined immunoturbidometrically with sheep anti-rabbit apoA-I polyclonal antibodies^16^. Total cholesterol concentrations were determined enzymatically^17^. Plasma HDL-C levels were determined after polyethylene glycol 6000 precipitation of apoB-containing lipoproteins^18^. Analyses were carried out using an AU480 Chemistry Analyzer (Beckman Coulter, Fullerton, CA).

### Assessment of neointimal hyperplasia in the grafted veins

The animals were euthanised 4 weeks after the vein grafting procedure. The grafted veins (~3 mm) were collected, fixed with paraformaldehyde (4%, v/v), embedded in paraffin and then sectioned (5 µm) as described^19^. Morphology was assessed with Verhoeff’s hematoxylin stain. Adjacent sections of the grafted veins were immunostained with mouse anti-rabbit vascular cell adhesion molecule-1 (VCAM-1) (1:400), mouse anti-rabbit intercellular adhesion molecule-1 (ICAM-1) (1:200) monoclonal antibodies (both gifts from Dr. M.Cybulsky, University of Toronto)^19^, an anti-mouse proliferating cell nuclear antigen (PCNA) monoclonal antibody (dilution 1:200; Dako, Glostrup, Denmark), a mouse monoclonal antibody against alpha smooth muscle actin (α-SMC actin) (dilution 1:50; Abcam, Cambridge, UK), a mouse monoclonal antibody against macrophage clone RAM11 (RAM11) (dilution 1:200; Dako), and a mouse monoclonal antibody against CD18 (1:200) (AbD Serotec, Raleigh, NC). A polyclonal goat anti-mouse IgG-HRP (dilution 1:200, Dako) was used as a secondary antibody. The Horseradish Peroxidase (HRP)-3,3′ Diaminobenzidine (DAB) system (Envision Mouse Kit, Dako), and counterstaining with haematoxylin was used for visualization of the stained samples. Sections were imaged with a light microscope (Zeiss, Germany). Planimetry (Adobe Photoshop V6.0) was performed by tracing the area of the intima and media. Results are reported as total pixel numbers. Intimal hyperplasia was evaluated as the intima/media ratio. DAB staining was quantified with ImageJ software (http://rsb.info.nih.gov/ij/) using the polygon tool to quantify the total intima/media cross-sectional area and lumen circumference. The threshold for positive staining was defined by an independent observer that was blinded to the treatment. Positively stained areas were quantified by de-convolution. To account for variations in grafted vein size, the number of pixels staining positive for VCAM-1- and ICAM-1 was divided by the circumference of the lumen^19^. Total cell profiles and PCNA^+^ cells were quantified manually (40x magnification). All the samples were coded and analysed by a single operator that was blinded to the treatment.

### Assessment of endothelial function

Isometric tension experiments for grafted vein rings (~3 mm in length) were performed 4 h after the animals were euthanised. The rings were positioned in cold Krebs buffer solution (Sigma-Aldrich, St Louis, MO, Catalogue Number: K3753), aerated with 95% O_2_/5% CO_2_ and mounted in a Myobath (World Precision Instruments, Sarasota, FL, USA) containing 20 mL of Krebs buffer solution aerated at 37 °C with 95% O_2_/5% CO_2_^19^. The viability of the rings was confirmed by incremental constriction (2.5 g load) in the presence of phenylephrine (Sigma-Aldrich, Catalogue Number: P6126)^19^. After pre-constricting the rings to 80% maximal response, endothelium-dependent vasodilation was quantified in the presence of incremental doses (0.001–10 µmol/L) of acetylcholine (Sigma-Aldrich, Catalogue Number: A6625), and sodium nitroprusside (Sigma-Aldrich, Catalogue Number: 71778).

### Statistical analysis

Data are expressed as mean ± SEM. Differences between groups were evaluated using an unpaired Student’s *t* test. Between group differences in acetylcholine and sodium nitroprusside dose response curves were evaluated by one-way ANOVA for repeated-measures with Bonferroni corrections. All statistical tests were performed using GraphPad Prism software version 7.03 (GraphPad Software, Inc. San Diego, CA). Result are expressed as the mean ± SEM. A 2-tailed p < 0.05 was considered significant.

## Results

### Des-fluoro-anacetrapib treatment inhibits CETP activity and increases intimal hyperplasia in grafted veins in NZW rabbits

**Figure 1:**
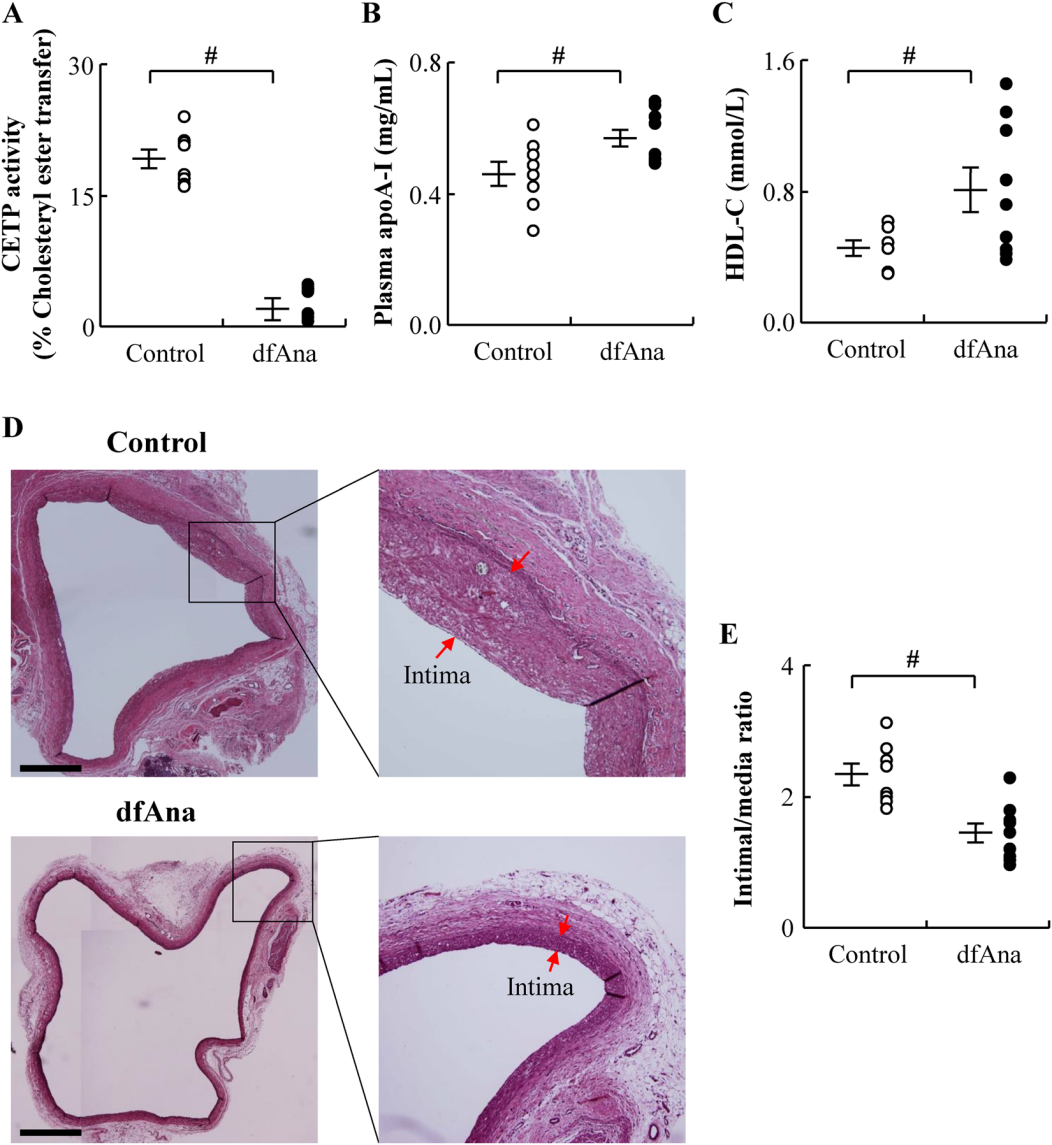
Dietary supplementation with des-fluoro-anacetrapib inhibits plasma CETP activity, and intimal hyperplasia in grafted veins in NZW rabbits. NZW rabbits received chow (control) or chow supplemented with 0.14% (wt/wt) des-fluoro-anacetrapib (dfAna) for 6 weeks. A right external jugular vein autologous end-to-side transplantation bypass graft was carried out after 2 weeks of des-fluoro-anacetrapib treatment. The animals were sacrificed 4 weeks after bypass grafting. Panel (A): plasma CETP activity. Panel (B): plasma apoA-I levels. Panel (C): plasma HDL-C levels. Panel (D): A representative Verhoeff’s hematoxylin-stained cross-section of the centre of a grafted vein (bar = 500 µm). Panel (E): Quantification of intima-to-media ratio of cross-sections of grafted veins. Data are expressed as individual points with the cross symbol indicating the mean ± SEM, n = 8 for the control group, n = 9 for the dfAna group, ^#^p < 0.05 vs Control.

Two groups of NZW rabbits (n = 8–9/group) were studied. Dietary supplementation with 0.14% (wt/wt) des-fluoro-anacetrapib reduced CETP activity by 89 ± 6.9% relative to the animals that were maintained on regular chow (Fig. 1A, p < 0.05). Plasma apoA-I levels increased from 0.46 ± 0.04 mg/mL for the control animals to 0.57 ± 0.03 mg/mL in the des-fluoro-anacetrapib-treated animals (Fig. 1B, p < 0.05). HDL-C levels increased from 0.42 ± 0.05 mmol/L in the control animals to 0.81 ± 0.14 mmol/L in the des-fluoro-anacetrapib-treated animals (Fig. 1C, p < 0.05).

We have reported elsewhere that des-fluoro-anacetrapib treatment inhibits intimal hyperplasia in NZW rabbits with balloon injury of the abdominal aorta^13^ and balloon injury and stent deployment in the iliac artery^14^. In the present study, right external jugular vein autologous end-to-side transplantation bypass grafting of the common carotid artery also led to neointimal formation in the grafted veins, as determined by the increased intima/media ratio, the control animals. (Fig. 1D, red arrows)). Grafted vein neointimal hyperplasia in the des-fluoro-anacetrapib-treated rabbits was, by contrast, decreased by 38 ± 6.2% compared to what was observed for the control animals (Fig. 1D,E, p < 0.05).

### Des-fluoro-anacetrapib treatment inhibits endothelial expression of VCAM-1 and ICAM-1 in grafted veins in NZW rabbits

**Figure 2:**
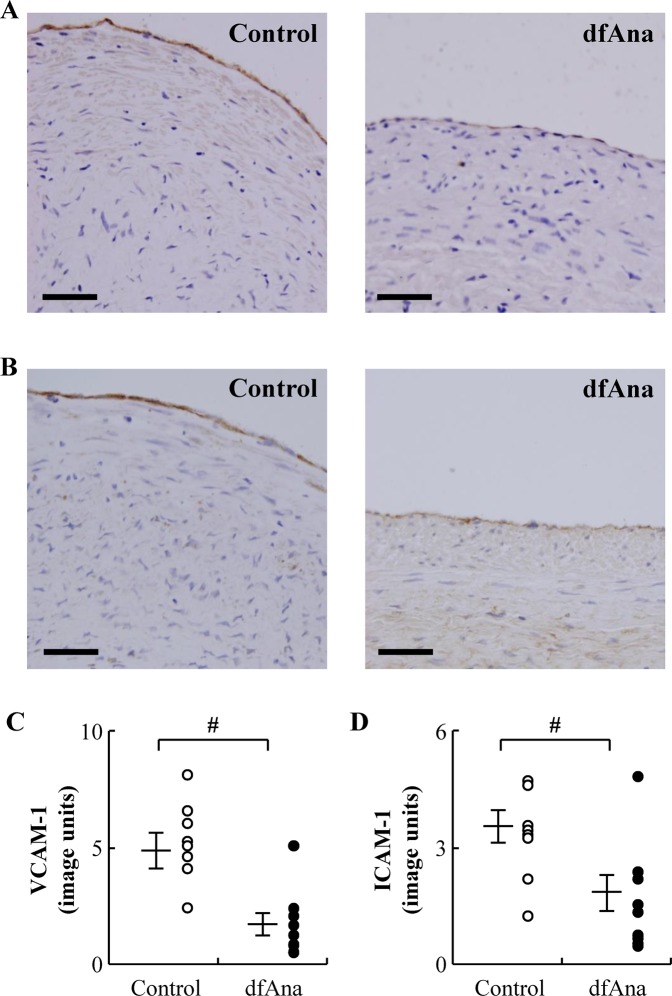
Des-fluoro-anacetrapib treatment decreases endothelial VCAM-1 and ICAM-1 expression in grafted veins in NZW rabbits. Right external jugular vein autologous end-to-side transplantation bypass grafting of the right common carotid artery was carried out in NZW rabbits maintained on regular chow (control) or chow supplemented with 0.14% (wt/wt) des-fluoro-anacetrapib (dfAna) as described in the legend to Fig. 1. VCAM-1 (Panel A) and ICAM-1 immunostaining (Panel B) of representative grafted vein cross-sections is shown (bar = 100 µm). Quantification of endothelial expression of VCAM-1 and ICAM-1 is shown in Panels (C,D), respectively. Data are expressed as individual points with the cross symbol indicating the mean ± SEM, n = 8 for the control group, n = 9 for the dfAna group, ^#^p < 0.05 vs Control.

The grafted veins in the control animals that did not receive des-fluoro-anacetrapib had high endothelial expression levels of VCAM-1 (Fig. 2A) and ICAM-1 (Fig. 2B). By contrast, endothelial expression of VCAM-1 (Fig. 2A) and ICAM-1 (Fig. 2B) in the des-fluoro-anacetrapib-treated rabbits was decreased by 65 ± 9.9% (Fig. 2C) and 51 ± 14% (Fig. 2D), respectively, compared to what was observed for the control animals (p < 0.05 for both).

### Des-fluoro-anacetrapib treatment reduces endothelial dysfunction in grafted veins in NZW rabbits

**Figure 3:**
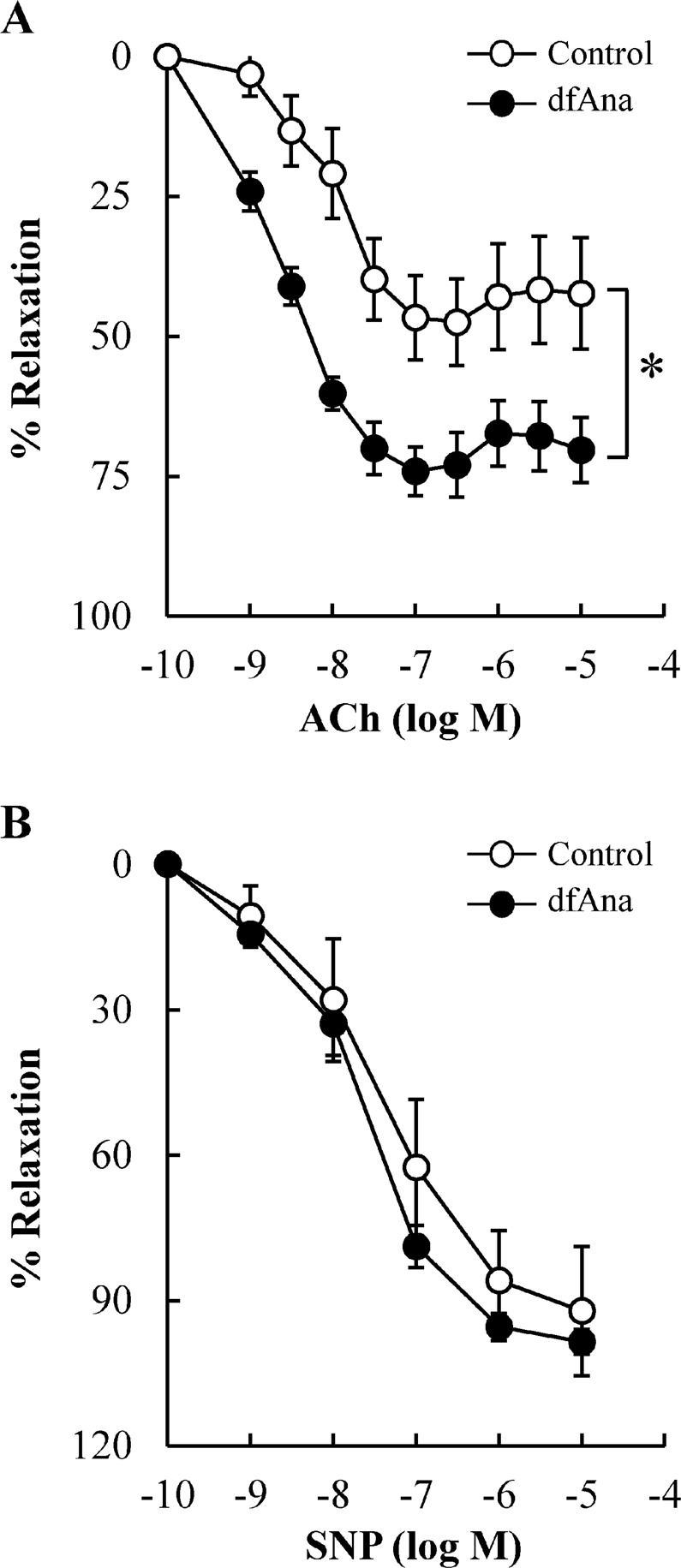
Des-fluoro-anacetrapib treatment protects against grafted vein endothelial dysfunction in NZW rabbits. Right external jugular vein autologous end-to-side transplantation bypass grafting of the right common carotid artery was carried out in NZW rabbits maintained on regular chow (control) or chow supplemented with 0.14% (wt/wt) des-fluoro-anacetrapib (dfAna) as described in the legend to Fig. 1. Panel (A): Endothelial-dependent relaxation of pre-contracted grafted vein rings in response to acetylcholine (ACh). Panel (B): Endothelial-independent relaxation of pre-contracted grafted vein rings in response to sodium nitroprusside (SNP). Data are expressed as mean ± SEM, n = 8 for the control group, n = 9 for the dfAna group, *p < 0.05.

Endothelial dysfunction was evident in the grafted veins in the control animals (Fig. 3A, open circles). Des-fluoro-anacetrapib treatment was associated with a maximal increase in endothelium-dependent relaxation in pre-constricted rings from the grafted veins in response to acetylcholine of 1.7 ± 0.2-fold relative to control (Fig. 3A, closed circles) (p < 0.05). Endothelium-independent relaxation with sodium nitroprusside was indistinguishable for the control and des-fluoro-anacetrapib-treated animals (Fig. 3B).

### Des-fluoro-anacetrapib treatment reduces smooth muscle cell proliferation in grafted veins in NZW rabbits

**Figure 4:**
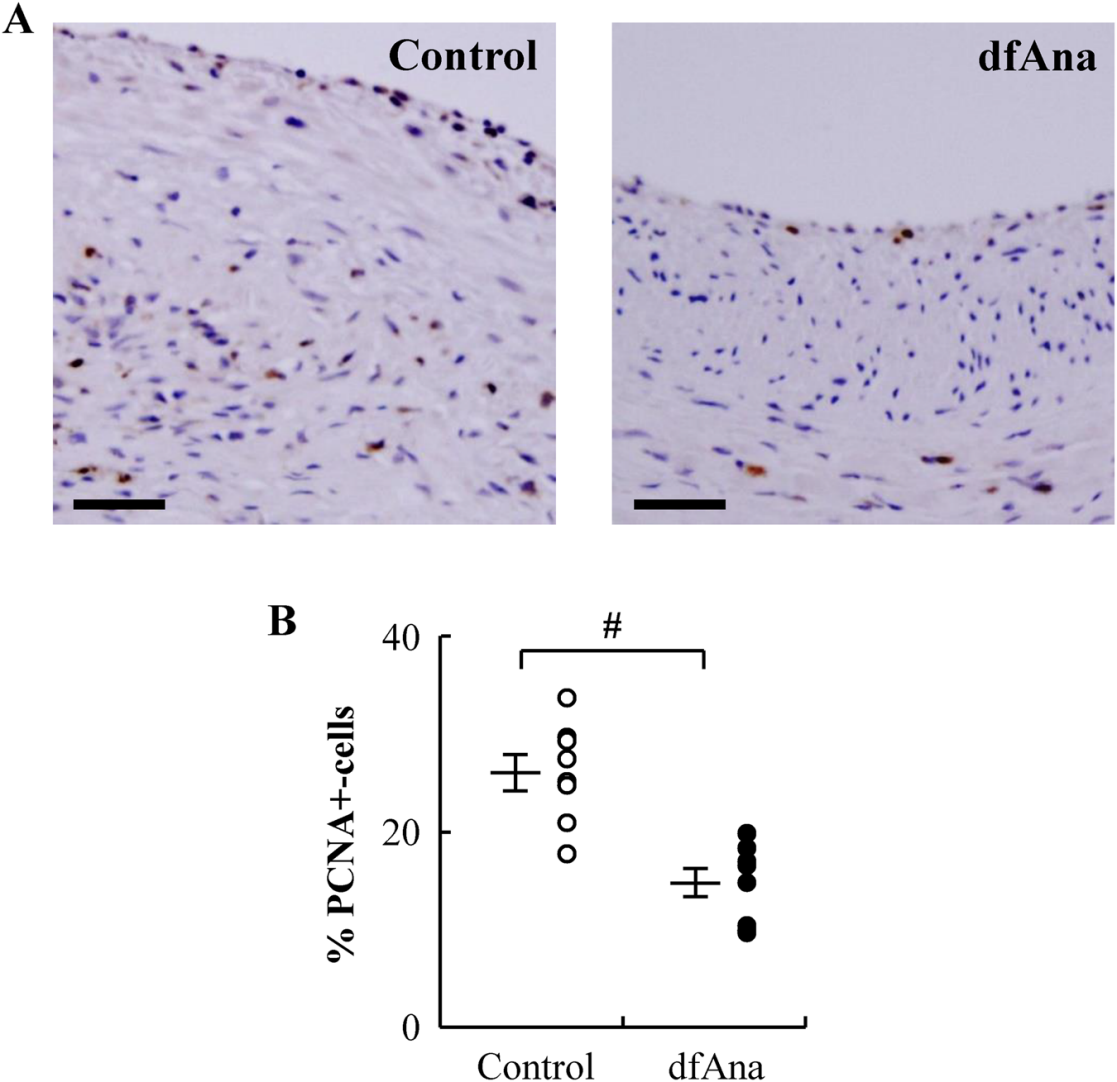
Des-fluoro-anacetrapib treatment reduces grafted vein smooth muscle cell proliferation in NZW rabbits. Right external jugular vein autologous end-to-side transplantation bypass grafting of the right common carotid artery was carried out in NZW rabbits maintained on regular chow (control) or chow supplemented with 0.14% (wt/wt) des-fluoro-anacetrapib (dfAna) as described in the legend to Fig. 1. Panel (A): immunostaining of PCNA^+^ cells in grafted vein cross-sections (bar = 50 µm). Panel (B): Quantification of cell proliferation expressed as the % PCNA^+^ cells relative to the total number of cells in the intima and media. Data are expressed as individual points with the cross symbol indicating the mean ± SEM, n = 8 for the control group, n = 9 for the dfAna group, ^#^p < 0.05 vs Control.

Smooth muscle cell (SMC) proliferation was apparent in the grafted veins of the control animals as judged by the numerous PCNA^+^ cells (Fig. 4A). Treatment with des-fluoro-anacetrapib was associated with a reduction in the number of PCNA^+^ cells in grafted veins of 43 ± 5.5% relative to what was observed for the control animals (Fig. 4B) (p < 0.05).

### Cellular characterization of the vein grafts

**Figure 5:**
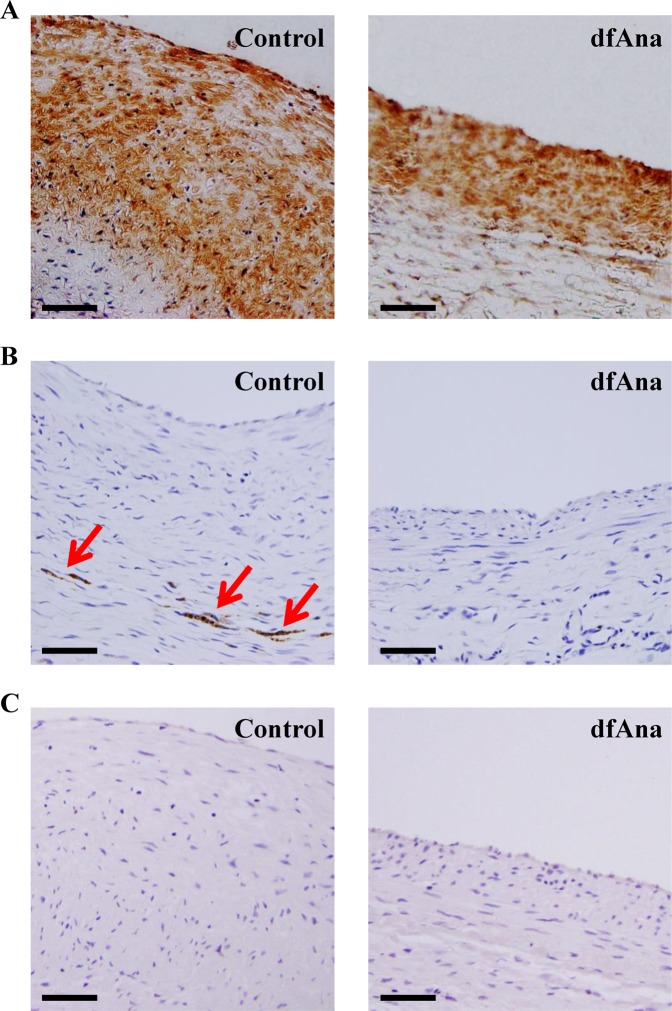
Cellular characterization of grafted veins in NZW rabbits treated with des-fluoro-anacetrapib. Right external jugular vein autologous end-to-side transplantation bypass grafting of the right common carotid artery was carried out in NZW rabbits maintained on regular chow (control, n = 8) or chow supplemented with 0.14% (wt/wt) des-fluoro-anacetrapib (dfAna, n = 9) as described in the legend to Fig. 1. Representative photomicrographs of grafted vein cross-sections (bar = 50 µm) from control and dfANa-treated animals immunostained for α-SMC actin (Panel A), CD18 (Panel B, red arrows) and RAM11 (Panel C) are shown.

As indicated by α-actin staining, the number of smooth muscle cells in the grafted veins of the control animals was increased relative to what was observed for the des-fluoro-anacetrapib-treated animals (Fig. 5A). A small number of neutrophils (CD18 + cells) were also evident in the neointimal region of the grafted veins (Fig. 5B). Macrophages (RAM11 + cells) were not detected in the grafted veins of the des-fluoro-anacetrapib-treated or control animals (Fig. 5C).

### Des-fluoro-anacetrapib treatment does not have a direct effect on vascular cell proliferation and inflammation

To ascertain whether the reduction in intimal hyperplasia in the des-fluoro-anacetrapib-treated animals could be attributed to a direct interaction of the inhibitor with intimal smooth muscle cells, human micro-vascular endothelial cells (HMECs) (Supplemental Fig. 1A) and human aortic smooth muscle cells (HASMCs) (Supplemental Fig. 1B) were incubated with des-fluoro-anacetrapib. Cell proliferation did not increase in either of these incubations (Supplemental Fig. 1). Moreover, incubation with des-fluoro-anacetrapib did not inhibit VCAM-1 (Supplemental Fig. 1C) or ICAM-1 (Supplemental Fig. 1D) mRNA levels in tumour necrosis factor (TNF)-α−activated HMECS.

## Discussion

Vein graft surgery has a high failure rate. Patency can decrease from 98% immediately after surgery to < 88% within the first month after surgery, and to 60% after 10 years as a result of intimal hyperplasia, atherosclerosis, and rupture of plaques in the vein grafts^20,21^. Anti-platelet agents and statins are the only medications currently used for prevention of vein graft failure^20^. As vein grafts will continue to be used in the foreseeable future, it is apparent that additional approaches for improving the patency of the grafted vessels are needed. The present study, which indicates that increasing HDL-C levels by inhibiting CETP activity is associated with reduced intimal hyperplasia in grafted veins, may be a further strategy for improving vein graft patency.

We have previously shown that increasing HDL-C levels with the CETP inhibitor des-fluoro-anacetrapib accelerates endothelial repair, enhances endothelial function, inhibits vascular SMC proliferation and reduces intimal hyperplasia in NZW rabbits with endothelial denudation of the abdominal aorta^13^ or balloon injury and stent deployment in the iliac artery^14^. In the previous studies, dietary supplementation with 0.14% (wt/wt) des-fluoro-anacetrapib increased plasma HDL-C levels and reduced CETP activity to a similar extent as in the present study. We have also reported that treating NZW rabbits with des-fluoro-anacetrapib increases HDL particle size, but does not affect plasma non-HDL-C or triglyceride levels^13,14^. It therefore follows that the protective effect of des-fluoro-anacetrapib against grafted vein neointimal formation is likely due to the increase in HDL-C levels.

Inflammatory responses, endothelial dysfunction and cellular proliferation are all associated with grafted vein neointimal formation^1,22–24^. In the present study smooth muscle cells were the dominant cell type in the grafted veins. A small number of neutrophils were also detected in the neointima. Although macrophages play an important role in vascular inflammation, they were not detected in the grafted veins, possibly because the animals were maintained on a normal chow diet for the duration of the study.

Our results indicate that the beneficial effects of dietary supplementation with des-fluoro-anacetrapib in terms of inhibiting endothelial cell inflammation decreasing expression of VCAM-1 and ICAM-1, reducing endothelial dysfunction and inhibiting smooth muscle cell proliferation in grafted veins are directly attributable to the increase in HDL-C levels in the des-fluoro-anacetrapib-treated animals and cannot be explained by an interaction of the inhibitor with smooth muscle cells or endothelial cells. These results are also in line with an exploratory analysis from the CASCADE trial in which lower HDL-C levels were associated with increased graft occlusion and intimal hyperplasia in the grafted veins^25^. Patients with HDL-C levels > 60 mg/dL also had significantly lower intimal hyperplasia at 12 months after coronary bypass surgery in that study^25^.

This may explain why there was reduced neointimal formation in the grafted veins of the des-fluoro-anacetrapib treated NZW rabbits. It is also possible that the reduced SMC proliferation and neointimal formation in the grafted veins of the des-fluoro-anacetrapib treated NZW rabbits may have occurred in an SR-B1/PDZK1- and PI3K/Akt-dependent manner as we have previously reported for des-fluoro-anacetrapib treated NZW rabbits with endothelial denudation of the abdominal aorta^13,14^.

HDLs and apoA-I are well-established independent inverse predictors of cardiovascular events. However, three large-scale randomized cardiovascular outcome clinical trials of CETP inhibitors have failed to meet their endpoints^26^. In the recent REVEAL trial, by contrast, anacetrapib significantly reduced major coronary events^27^. Although anacetrapib has a long terminal half-life because of accumulation in adipose tissue, and its development has stopped^28,29^, the results of the present study have identified a potential application of CETP inhibition as a therapy for preventing graft failure due to stenosis. Further studies in patients undergoing coronary artery bypass grafting are warranted.

There are some limitations in the present study. Only one dose of 0.14% (wt/wt) des-fluoro-anacetrapib was used, and we therefore cannot determine if there is a dose-dependent association of des-fluoro-anacetrapib with inhibition of neointimal formation in grafted veins. However, in our previous studies the effects of 0.14% (wt/wt) des-fluoro-anacetrapib on plasma HDL-C levels, CETP activity, angiogenesis in hindlimb ischemia, and endothelial function and repair were larger than those of 0.07% (wt/wt) des-fluoro-anacetrapib. It is therefore likely that des-fluoro-anacetrapib will also protect against grafted vein neointimal formation in a dose-dependent manner. Moreover, as the present study was performed in NZW rabbits, further studies are needed to determine whether CETP inhibition also decreases neointimal formation in grafted veins in humans. A further limitation of this study is that neointimal hyperplasia and endothelial function were evaluated only at the single time point of 4 weeks after the vein graft procedure. This precluded investigation of acute inflammatory cell recruitment and pro-inflammatory cytokine and chemokine production in the first few days after the procedure that could contribute to early vein graft failure^30,31^.

In conclusion, inhibition of CETP activity inhibits intimal hyperplasia in grafted veins in NZW rabbits by inhibiting endothelial inflammation, improving endothelial function, and reducing SMC proliferation. Further clinical studies are needed to determine whether CETP inhibition, or other HDL raising agents, reduce the incidence of graft failure due to stenosis in humans.

## Supplementary information

Supplemental Material
