# Supplementary material for: The Cholesteryl Ester Transfer Protein Inhibitor, des-Fluoro-Anacetrapib, Prevents Vein Bypass-induced Neointimal Hyperplasia in New Zealand White Rabbits
Source: Sci Rep. 2019 Nov 7;9:16183. doi: 10.1038/s41598-019-52510-0 (PMC6838195; doi:10.1038/s41598-019-52510-0)
Supplement: Supplementary file 1 — Supplemental Material [file 41598_2019_52510_MOESM1_ESM.pdf]

SUPPLEMENTAL MATERIAL

The Cholesteryl Ester Transfer Protein Inhibitor, des-Fluoro-Anacetrapib, Prevents Vein Bypass-induced Neointimal Hyperplasia in New Zealand White Rabbits

Ben J. Wu[a,*], Yue. Li[a], Kwok-Leung Ong[a], Yidan Sun[b], Douglas Johns[c], Philip J. Barter[a], Kerry-Anne Rye[a,*]

[a]Lipid Research Group, School of Medical Sciences, The University of New South Wales Sydney, New South Wales, Australia

[b]Otto Loewi Research Center for Vascular Biology, Immunology and Inflammation, Immunology and Pathophysiology, Medical University of Graz, Austria

[c]Merck & Co., Inc, Kenilworth, NJ, USA

*Correspondence authors:

Professor Kerry-Anne Rye or Dr Ben Wu, School of Medical Sciences, Faculty of Medicine, University of New South Wales Sydney, New South Wales, Australia 2052. Phone: (612) 9385-1219, Fax: (612) 9385-1389. E-mail: k.rye@unsw.edu.au or ben.wu@unsw.edu.au

Materials and Methods

Cell culture

Human micro-vascular endothelial cells (HMECs) (American Type Culture Collection, Manassas, VA, Catalogue Number: CRL-3243) and human aortic smooth muscle cells (HASMCs) (Cell Applications, San Diego, CA, Catalogue Number: 354-05a) were cultured at 37 °C in MCDB 131 medium (Sigma-Aldrich, St Louis, MO, Catalogue Number: M8537) and in Waymouth's MB752/1 medium (Sigma-Aldrich, Catalogue Number: W1625), respectively, in a 5% CO_2 incubator.

HMECs and HASMCs were grown to 50% confluence. The culture medium was replaced with medium without (control) or with des-fluoro-anacetrapib (dfAna) (final concentration 1 μM/L) and the cells were incubated for a further 24 h. Cell proliferation was assessed by Trypan blue exclusion and counting of total cells using a hemocytometer. In other experiments the cells were treated without or with df-Ana for 18 h, then stimulated with TNF-α (final concentration 1 ng/mL) for a further 6 h.

qPCR

Total RNA was isolated from cells using an RNeasy Mini Kit (Qiagen, Chadstone Centre, VIC, Australia, Catalogue Number: 74104). cDNA was synthesized using iScript Reverse Transcription Reagents (Bio-Rad, Gladesville, NSW, Australia; Catalogue number 170-8840). Target gene amplification was performed using iQ SYBR Green Super mix (BioRad; Catalogue number 170-8880) and run in sets of three replicates using a CFX96 real-time PCR detection system (Bio-Rad). The average threshold cycle (CT) was determined from triplicate reactions. Relative mRNA quantification was calculated by the 2-(ΔCT) method. The results were invariant irrespective of whether they were normalized to ß-actin. The PCR primers were: human β-actin: Sense 5’-GATCGCTGACCGTATGCAG-3’, Antisense 5’-

GTCGTACTCCTGCTTGGTG-3’; human VCAM-1: Sense 5’-ATGTAGTGTCATGGGCTGTG-3’, Antisense: 5’-GGAATGAGTAGAGCTCCACC-3’, and human ICAM-1: Sense 5’-CCATCTACAGCTTTCCGGCGC-3’, Antisense: 5’-CTCTGGGGTGGCCTTCAGCA-3’.

Statistical analysis

Data are expressed as mean ± SEM. An unpaired student’s *t* test was used to evaluate differences between groups. All statistical analyses were performed using GraphPad Prism software version 7.03 (GraphPad Software, Inc. San Diego, CA) and are expressed as the mean±SEM. A 2-tailed $p<0.05$ was considered significant.

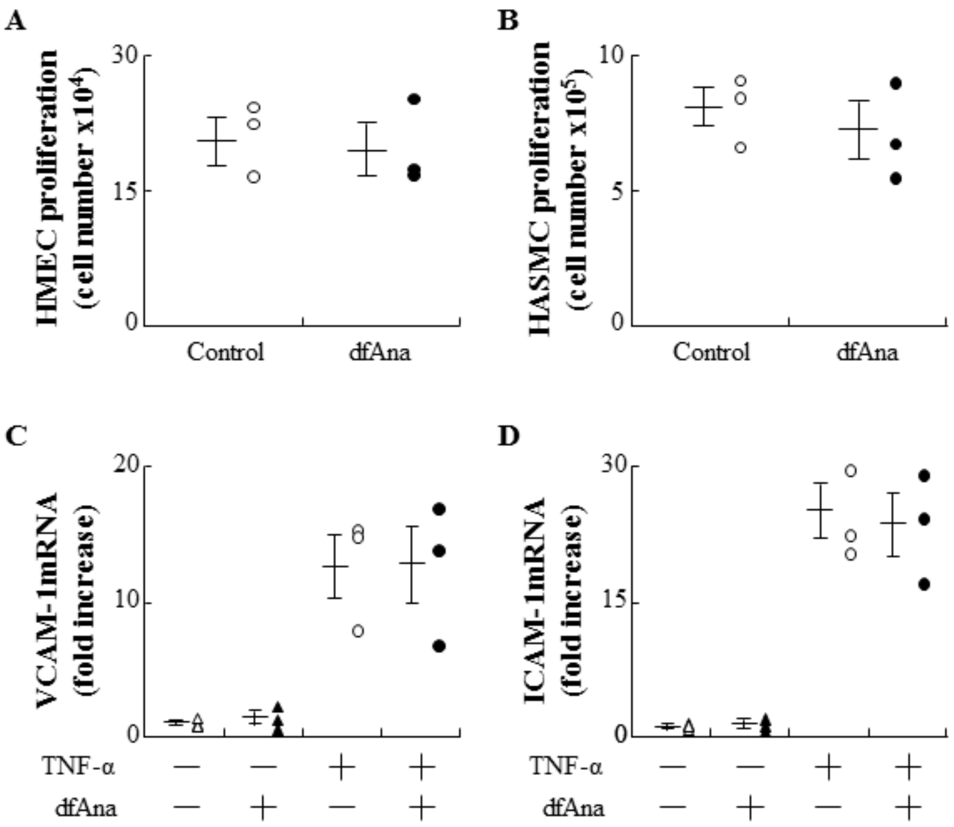

Supplemental Fig. I. Des-fluoro-anacetrapib does not affect vascular endothelial cell and smooth muscle cell proliferation or endothelial cell inflammatory responses. HMECs and HASMCs were incubated at 37 °C for 24 h in the presence or absence of dfAna (final concentration 1 μM/L) as described in Materials and Methods. Cell proliferation was assessed by Trypan Blue exclusion in HMECs (**Panel A)** and HASMCs (**Panel B)**. HMECs were incubated for 18 h in the absence or presence of dfAna (final concentration 1 μM), then stimulated for 6 h with TNF-α (final concentration 1 ng/mL). VCAM-1 (**Panel C**) and ICAM-1 mRNA levels (**Panel D)** were quantified by qPCR. Data are expressed as the mean±SEM of three independent experiments.
